# High-Density Genome-Wide Association Mapping Identifies Candidate Loci Associated with Maize Stalk Cell Wall Composition

**DOI:** 10.3390/plants15172696

**Published:** 2026-09-02

**Authors:** Yuan Ren, Jin Zhang, Qijian Tian, Xin Liu

**Affiliations:** 1 Center for Agricultural Genetic Resources Research, Shanxi Agricultural University, Taiyuan 030031, China; 2 Shanxi Functional Food Research Institute, Shanxi Agricultural University, Taiyuan 030031, China

**Keywords:** maize, genome-wide association study, cell wall composition, lignin, cellulose, hemicellulose, haplotype analysis, selective sweep

## Abstract

Maize (*Zea mays* L.) stalk cell wall composition is a key determinant of forage digestibility, lodging resistance, and biomass utilization efficiency. Although previous genome-wide association studies (GWAS) have identified loci associated with lignin (LIG), cellulose (CEL), and hemicellulose (HC), advances in genomic resources provide an opportunity to revisit existing phenotypic datasets at substantially higher resolution. Here, we re-analyzed a maize association panel consisting of 341 diverse inbred lines using an expanded genotype dataset containing 10.77 million SNPs, two derived compositional indices (CEL/HC and [LIG/(CEL + HC)], and six complementary GWAS models. Across all traits and models, we identified 855 unique significant SNPs associated with 579 candidate genes. Among the traits examined, LIG/(CEL + HC) yielded the greatest number of associations, suggesting that indices representing the relative balance among cell wall components may better capture the genetic architecture of cell wall composition than individual component measurements alone. Integration of multiple GWAS models with functional enrichment, haplotype, and selective sweep analyses prioritized three biologically relevant candidate genes encoding a MYB58 transcription factor, the glycosyltransferase Xt9, and a putative xyloglucan 6-xylosyltransferase. Haplotype analysis revealed significant effects of *Xt9* and the xyloglucan 6-xylosyltransferase on cell wall composition, while selective sweep analysis identified *Xt9* as a target of repeated selection during maize domestication, ecological adaptation, and modern breeding. Although these candidate genes provide promising targets for future investigation, the associations identified here are based on a single association panel and require functional and independent population validation. Collectively, our results demonstrate how high-density genotyping combined with complementary GWAS models can refine candidate associations and generate testable hypotheses from existing phenotypic datasets.

## 1. Introduction

Maize (*Zea mays* L.) is a globally important staple crop whose stover serves as a primary energy source for ruminant livestock and a promising feedstock for lignocellulosic biofuel production [1,2]. The feeding value and industrial utility of maize stover are largely determined by three major cell wall components—lignin (LIG), cellulose (CEL), and hemicellulose (HC)—which collectively influence stalk strength, lodging resistance, pathogen defense, and fiber digestibility [3,4,5,6]. Understanding the genetic architecture underlying these cell wall constituents is therefore essential for both silage maize improvement and the development of dual-purpose varieties that balance agronomic performance with biomass quality.

Quantitative trait locus (QTL) mapping in bi-parental populations has identified numerous genomic regions associated with maize cell wall composition [7,8,9,10,11,12,13,14]. However, the limited recombination events and low marker density inherent to linkage mapping restrict both mapping resolution and allele coverage. Genome-wide association studies (GWAS) overcome these limitations by exploiting historical recombination in diverse panels, enabling higher-resolution dissection of complex traits [15]. We previously performed the first GWAS specifically focused on the three major components of maize stalk cell walls using an association panel of 368 inbred lines evaluated across seven environments, with approximately 0.56 million single nucleotide polymorphisms (SNPs) at a minor allele frequency (MAF) threshold of 0.05 [16]. That study identified 22, 18, and 24 significantly associated loci for LIG, CEL, and HC, respectively, with individual loci explaining 4–7% of phenotypic variation. Candidate genes at these loci predominantly encoded enzymes in cell wall metabolism, transcription factors, and protein kinases [16].

Despite these advances, the original analysis employed a single statistical model and a marker density that is modest by current sequencing standards. Since that study, whole-genome resequencing, genotype imputation, and improved reference genomes have dramatically increased marker density available for association analyses in maize and other crop species. Higher marker density increases the likelihood that causal variants or tightly linked markers are captured, particularly in regions of rapid linkage disequilibrium decay [17], while improved analytical methods provide complementary approaches for detecting loci with different genetic architectures. Because individual GWAS models differ in their treatment of population structure, relatedness, and multi-locus effects, they frequently identify overlapping but non-identical sets of significant loci [18,19]. Loci consistently detected across models with complementary statistical foundations are more likely to represent true biological signals, whereas model-specific associations may reflect false positives or artifacts of a particular modeling framework.

Here, we reanalyzed the original association panel of 341 maize inbred lines using previously generated phenotypic data for LIG, CEL, and HC, together with two derived compositional indices and an expanded genotype dataset comprising 10.77 million SNPs. To improve the robustness of locus detection, we applied six complementary GWAS models—GLM, MLM, MLMM, SUPER, BLINK, and FarmCPU [20,21,22,23,24,25]—and integrated the resulting associations with functional enrichment, haplotype and selective sweep analyses. This integrated analytical framework enabled us to prioritize candidate genes for further investigation, evaluate the consistency of association signals across models in the context of increased marker density, and generate testable hypotheses regarding the genetic architecture of maize stalk cell wall composition. We emphasize that all associations reported here are statistical in nature and require independent validation in additional populations and through functional assays.

## 2. Results

### 2.1. Expanded Marker Data and Phenotypic Variation

**Figure 1:**
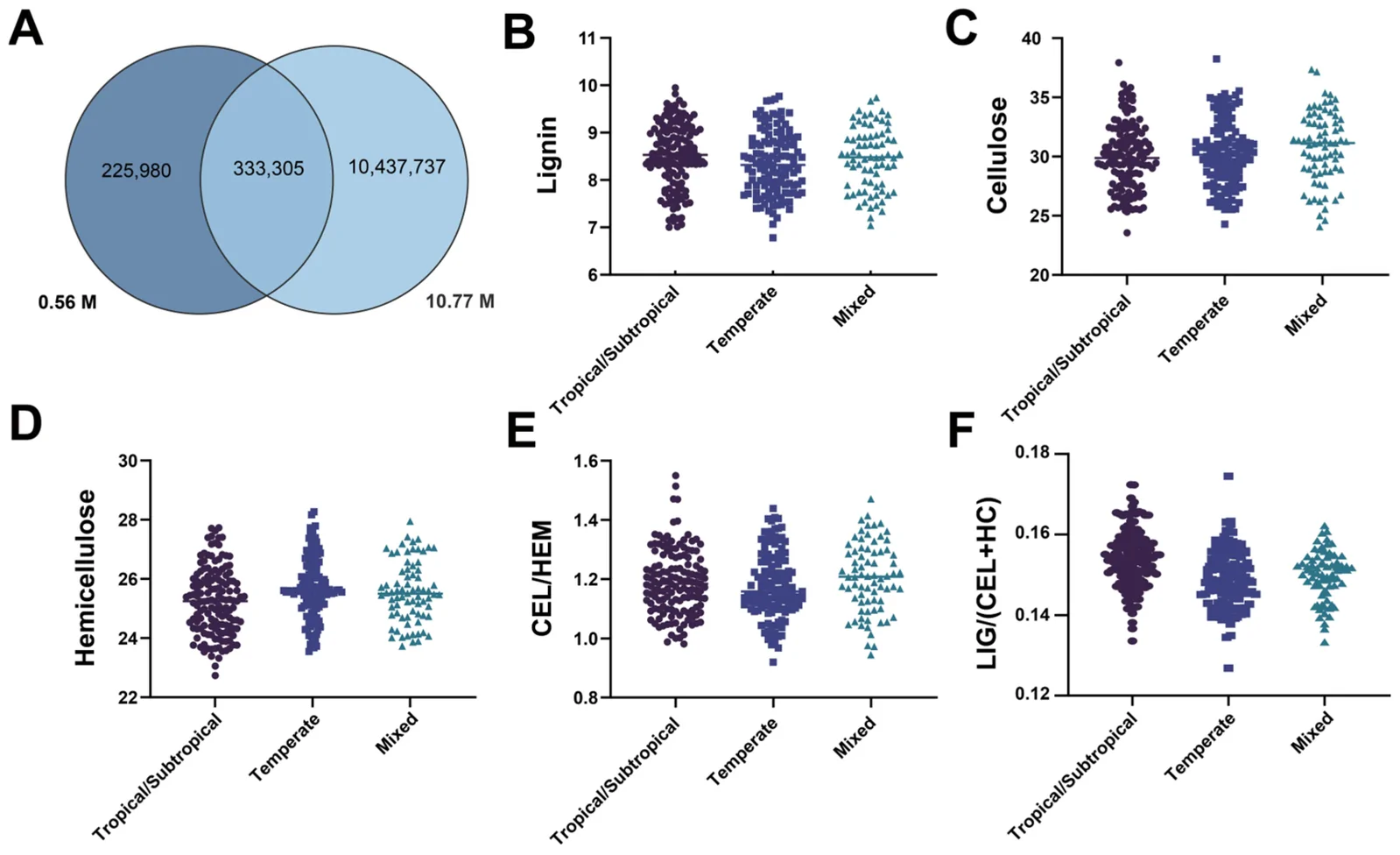
Phenotypic distribution of cell wall components across 341 maize inbred lines and comparison of marker density. (**A**) Comparison of marker density between the high-density 10.77 M panel and the standard 0.56 M panel. (**B**–**F**) Frequency distributions of lignin (LIG), cellulose (CEL), hemicellulose (HC), CEL/HEM ratio, and LIG/(CEL + HC) ratio, respectively. Different colors represent distinct subpopulations, including tropical/subtropical, temperate SS, temperate NSS, and mixed groups.

To extend our previous genome-wide association study of maize stalk cell wall composition, we reanalyzed the same diverse panel of 341 maize inbred lines using an expanded marker dataset comprising 10.77 million SNPs, including more than 10.4 million newly introduced markers (Figure 1A). This substantial increase in marker density improved genome coverage, reduced gaps between adjacent markers, and enabled more precise delineation of linkage disequilibrium blocks, thereby providing a stronger basis for the subsequent fine-mapping of causal variants. In addition to the three primary cell wall components analyzed previously, namely lignin (LIG), cellulose (CEL), and hemicellulose (HC) contents, we also evaluated two derived compositional indices, the cellulose-to-hemicellulose ratio (CEL/HC) and the lignin-to-structural carbohydrate ratio [(LIG/(CEL + HC)] to capture the relative balance among major cell wall constituents. The CEL/HEM ratio ranged between 0.92 and 1.55 (mean = 1.19 ± 0.11), whereas the LIG/(CEL + HC) ratio spanned from 0.127 to 0.174 (mean = 0.151 ± 0.007). All five traits exhibited substantial phenotypic variation and approximately normal distributions across the population, with broadly similar distributions observed among the tropical/subtropical, temperate (Stiff Stalk [SS] and Non-Stiff Stalk [NSS]), and mixed subpopulations (Table S2; Figure 1B–F).

### 2.2. Genome-Wide Association Mapping

Genome-wide association analyses were then performed using six complementary GWAS models to identify loci associated with the five cell wall-related traits. Model diagnostics indicated adequate control of population structure and kinship, and numerous significant associations were detected across all traits (Supplementary Figures S1 and S2). Because the six GWAS models differ in their statistical assumptions and detection power, associations were identified independently with each model to improve the robustness of locus detection. Accordingly, the number of significant associations varied among models, ranging from 17 to 721 significant SNPs and from 34 to 398 candidate genes (Table S3). Significant SNPs identified across the six models and their corresponding candidate genes were then consolidated into nonredundant datasets comprising 855 unique SNPs and 579 unique genes for subsequent analyses. Of the unique SNPs identified across all models, 150 were detected by at least two models, including 24 supported by three or more models and three identified by four models (GLM, SUPER, FarmCPU, and MLMM). Among the five traits, the derived LIG/(CEL + HC) ratio yielded the greatest number of associations (599 SNPs and 110 candidate genes), followed by LIG, CEL, CEL/HC, and HC (Table S3).

### 2.3. Comparison of QTL Detection Between the 0.55 M and 10.77 M Marker Sets

**Figure 2:**
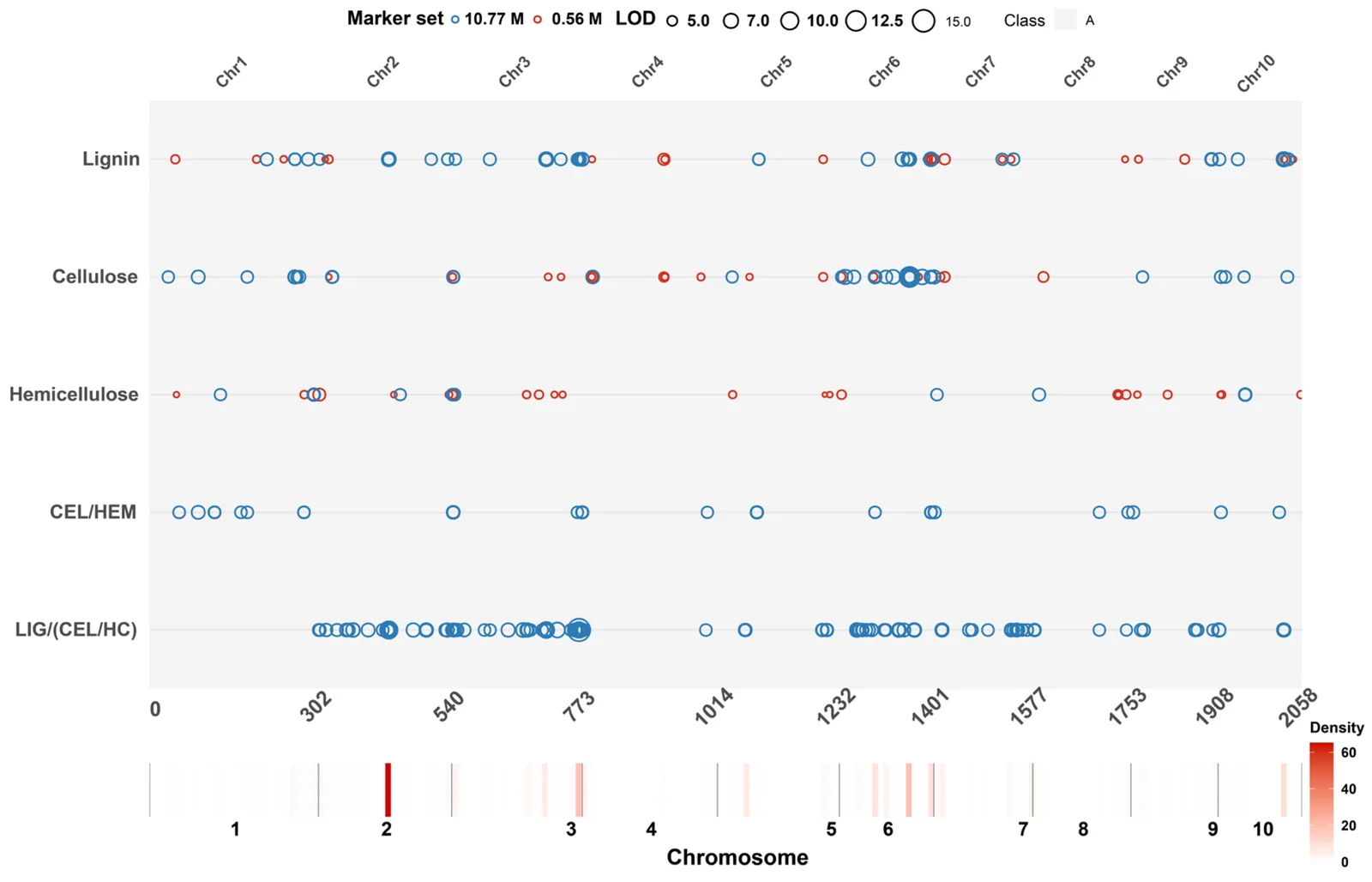
Chromosomal distribution and co-localization of candidate loci identified in the previous and current GWAS for maize stalk cell wall composition. The outer tracks represent the 10 maize chromosomes. Candidate loci identified in the current high-density GWAS (10.77 M SNPs) are shown in blue, whereas loci identified in the previous study (559,285 SNPs) are shown in red. Overlapping genomic regions indicate co-localized loci detected in both studies. The figure summarizes the genome-wide distribution of candidate loci and the extent of overlap between the two GWAS analyses.

To evaluate the effect of marker density on QTL detection, we compared the GWAS results obtained using the 0.55 M and 10.77 M marker sets (Figure 2; Table S4). Across all traits, 64 QTL were detected with the 0.55 M marker set, whereas 855 QTL were identified with the expanded 10.77 M marker set. For the three traits analyzed using both marker sets, the number of detected QTL increased from 18 to 131 for cellulose and from 22 to 216 for lignin, whereas the number detected for hemicellulose decreased from 24 to 15. Overall, the expanded marker set substantially increased the number and genome-wide distribution of detected loci, particularly for lignin and cellulose. In addition, 38 and 599 QTL were identified for CEL/HC and LIG/(CEL + HC), respectively, using the 10.77M marker set. Comparison of genomic positions showed that most loci detected with the two marker sets were distinct, although several genomic regions were identified by both datasets. These results indicate that the expanded marker set retained some previously detected association signals while identifying additional candidate loci.

### 2.4. Functional Enrichment and Haplotype Analyses Prioritize Candidate Genes

**Figure 3:**
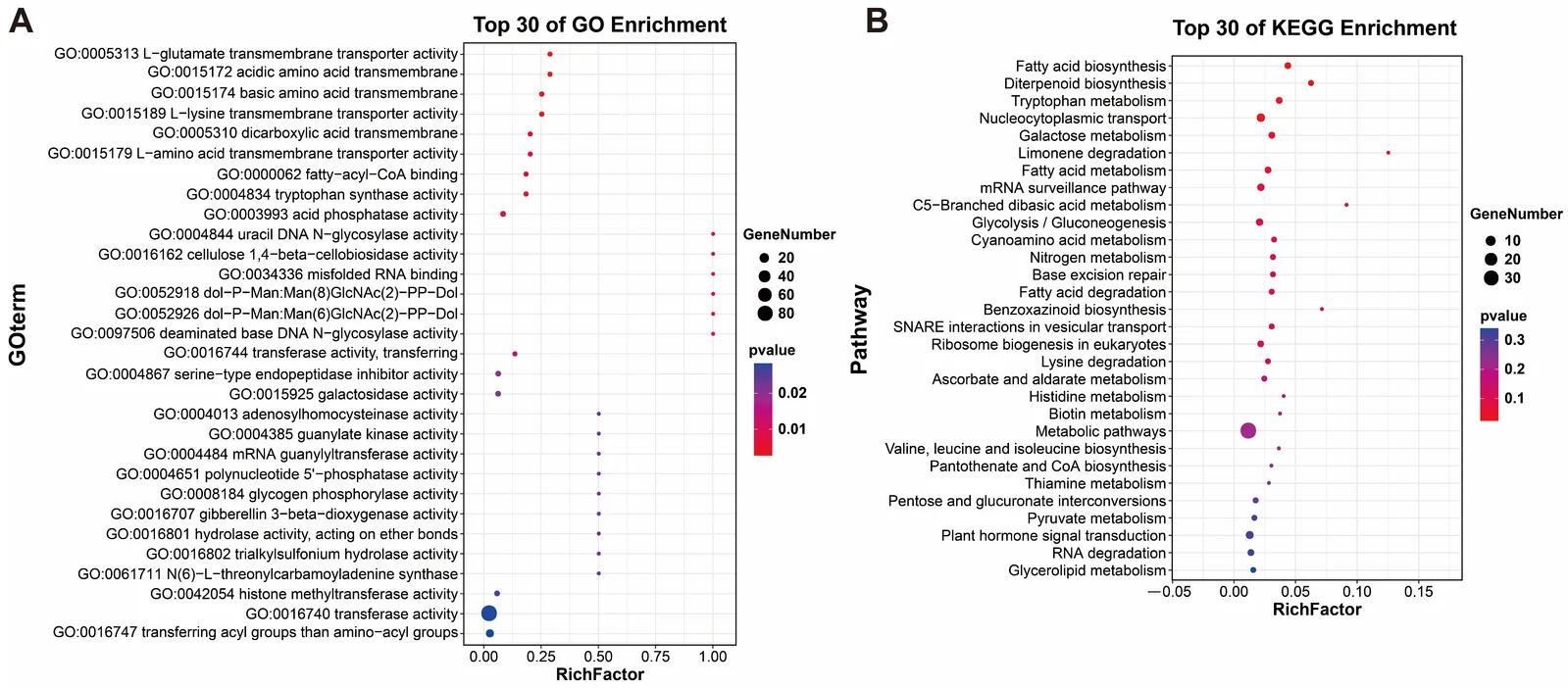
Functional enrichment analysis of candidate genes identified through GWAS. (**A**) Gene Ontology (GO) enrichment analysis of the 279 unique candidate genes, showing significantly overrepresented Molecular Function categories. (**B**) Kyoto Encyclopedia of Genes and Genomes (KEGG) pathway enrichment analysis revealing the top enriched biochemical pathways.

Gene Ontology (GO) and KEGG enrichment analyses were performed on the 579 unique candidate genes to identify biological processes associated with maize cell wall composition. GO analysis revealed significant enrichment of transferase activity and related functions involved in polysaccharide metabolism such as acyl group transferase activity and mannosyltransferase activity (Table S5, Figure 3A). Similarly, KEGG analysis identified enrichment of pathways associated with fatty acid biosynthesis, diterpenoid biosynthesis, and galactose metabolism (Table S6, Figure 3B). These enrichment analyses indicate that the identified candidate genes are enriched for functions associated with cell wall biosynthesis and metabolism.

**Figure 4:**
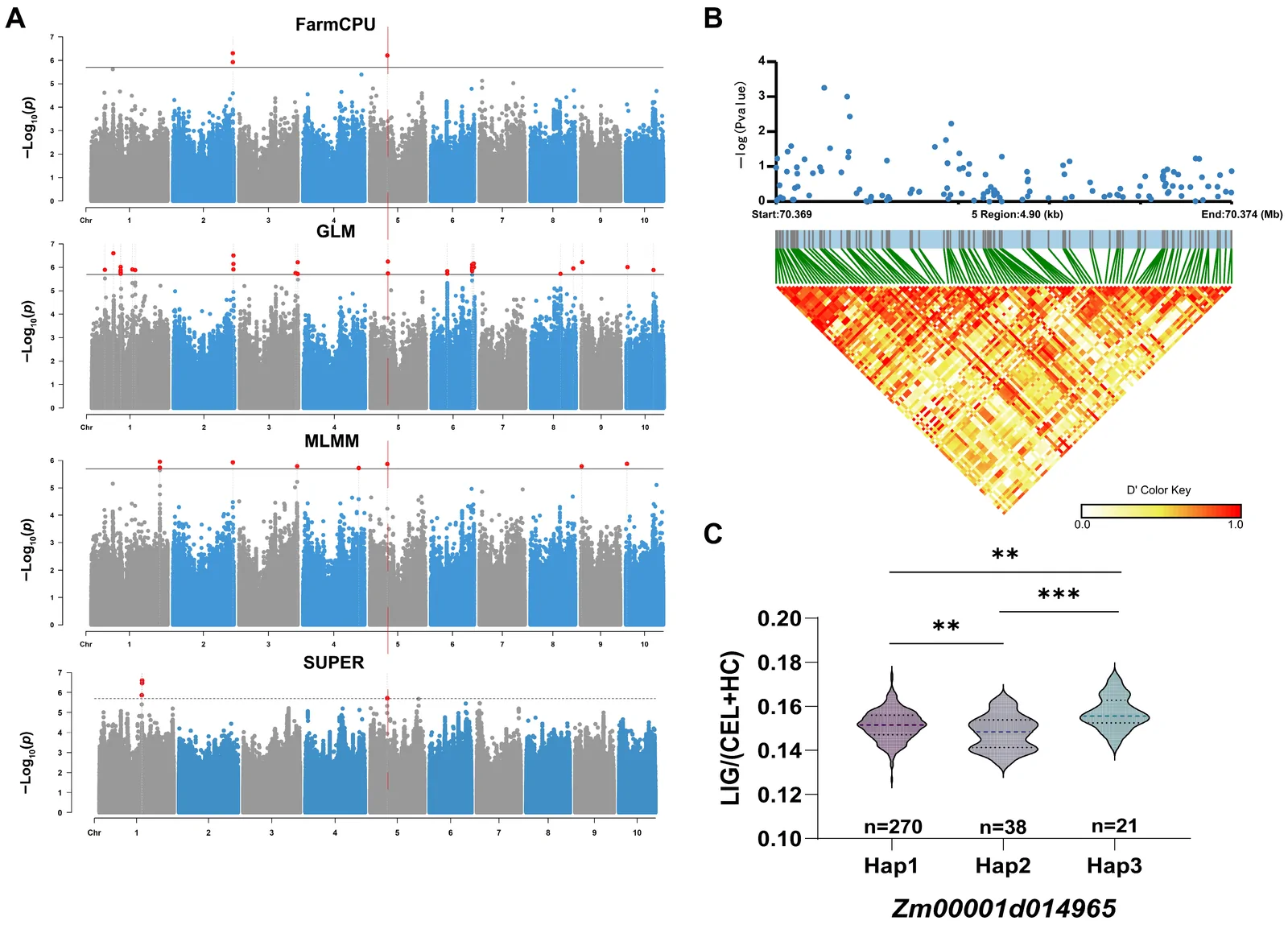
Association mapping and haplotype analysis of *Zm00001d014965*. (**A**) Manhattan plots showing the GWAS signals for *Zm00001d014965* detected by four models (FarmCPU, GLM, MLMM, and SUPER). (**B**) Linkage disequilibrium (LD) heatmap of the genomic region encompassing *Zm00001d014965*, with pairwise R^2^ values indicating strong LD blocks (R^2^ > 0.8). (**C**) Haplotype–phenotype association analysis of *Zm00001d014965*, comparing cell wall traits among distinct allelic groups. ** and *** indicate significant differences at *p* < 0.01 and *p* < 0.001, respectively.

**Figure 5:**
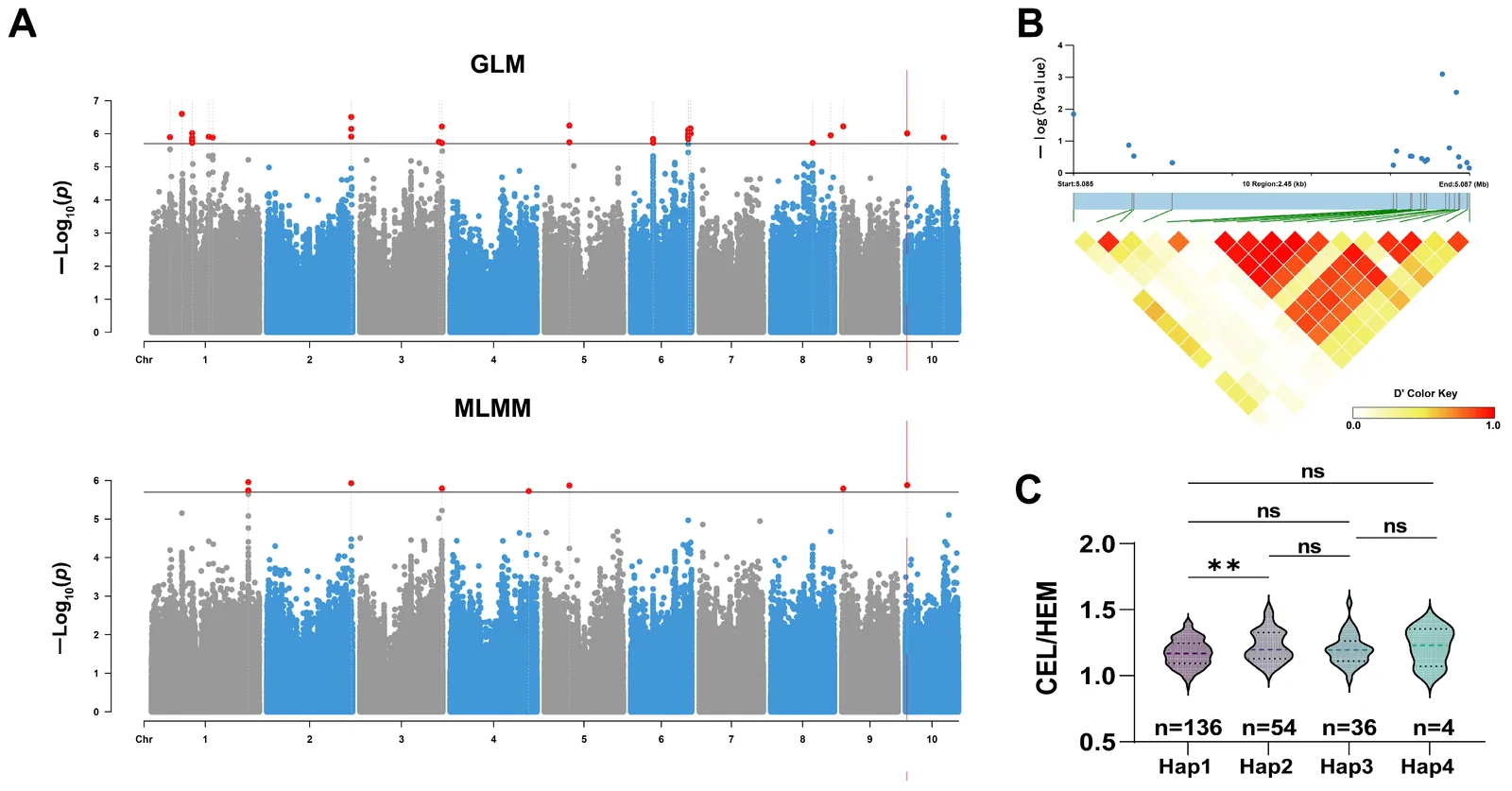
Association mapping and haplotype analysis of Zm00001d023417. (**A**) Manhattan plots showing the GWAS signals for Zm00001d023417 detected by multiple models (GLM and MLMM). (**B**) Linkage disequilibrium (LD) heatmap of the genomic region encompassing Zm00001d023417, with pairwise R^2^ values indicating strong LD blocks (R^2^ > 0.8). (**C**) Haplotype–phenotype association analysis of Zm00001d023417, comparing cell wall traits among distinct allelic groups. ** indicates a significant difference at *p* < 0.01, whereas ns indicates no significant difference (*p* ≥ 0.05).

Based on cross-model support and functional annotations related to cell wall biosynthesis, three candidate genes associated with the LIG/(CEL + HC) ratio were prioritized for further analyses. *Zm00001d002476* encodes a MYB58 transcription factor whose Arabidopsis ortholog, AtMYB58, is a well-characterized transcriptional regulator of lignin biosynthesis [26]. Beta-1,2-xylosyltransferase9 (*Xt9*; *Zm00001d014965*) encodes a glycosyltransferase involved in the synthesis of cell wall polysaccharides and was detected by all four active GWAS models, representing the strongest cross-model candidate identified in this study (Figure 4A). *Zm00001d023417* encodes a xyloglucan xylosyltransferase belonging to an enzyme class involved in hemicellulose biosynthesis and cell wall assembly, which was detected by GLM and MLMM models (Figure 5A).

To further evaluate the three prioritized candidate genes, linkage disequilibrium (Figure 4B and Figure 5B) and haplotype analyses (Figure 4C and Figure 5C) were performed. The MYB58 ortholog, *Zm00001d002476*, did not show significant differences in cell wall phenotypes among haplotypes. This result suggests that the sequence variation captured at *Zm00001d002476* is not a major determinant of cell wall phenotypic variation in the present association panel, although additional functional variants or regulatory polymorphisms at this locus cannot be excluded. In contrast, *Xt9* and *Zm00001eb406590* (encoding a probable xyloglucan xylosyltransferase) exhibited significant haplotype effects across multiple cell wall traits. For both genes, significantly associated SNPs were located within regions of strong linkage disequilibrium, supporting the presence of linked functional variation within these loci (Figure 4 and Figure 5).

For *Xt9*, three major haplotypes, Hap1 (n = 270), Hap2 (n = 38) and Hap3 (n = 21), were identified. Lines carrying Hap3 exhibited a significantly higher LIG/(CEL + HC) ratio than those carrying Hap1 and Hap2, accompanied by a relative reduction in hemicellulose content (Figure 4C, Table S7). Four major haplotypes were identified for *Zm00001d023417*: Hap1 (n = 136), Hap2 (n = 54), Hap3 (n = 36) and Hap4 (n = 4). Hap1 exhibited a significantly lower CEL/HC ratio than Hap2 (Figure 5C, Table S8), indicating that allelic variation at this locus influences the relative abundance of cellulose and hemicellulose. Collectively, these findings demonstrate that natural variation in *Xt9* and *Zm00001d023417* is significantly associated with maize cell wall composition, supporting these genes as strong candidates for future functional validation.

### 2.5. Selective Sweep Analyses Reveal Contrasting Evolutionary Histories of Candidate Genes

**Figure 6:**
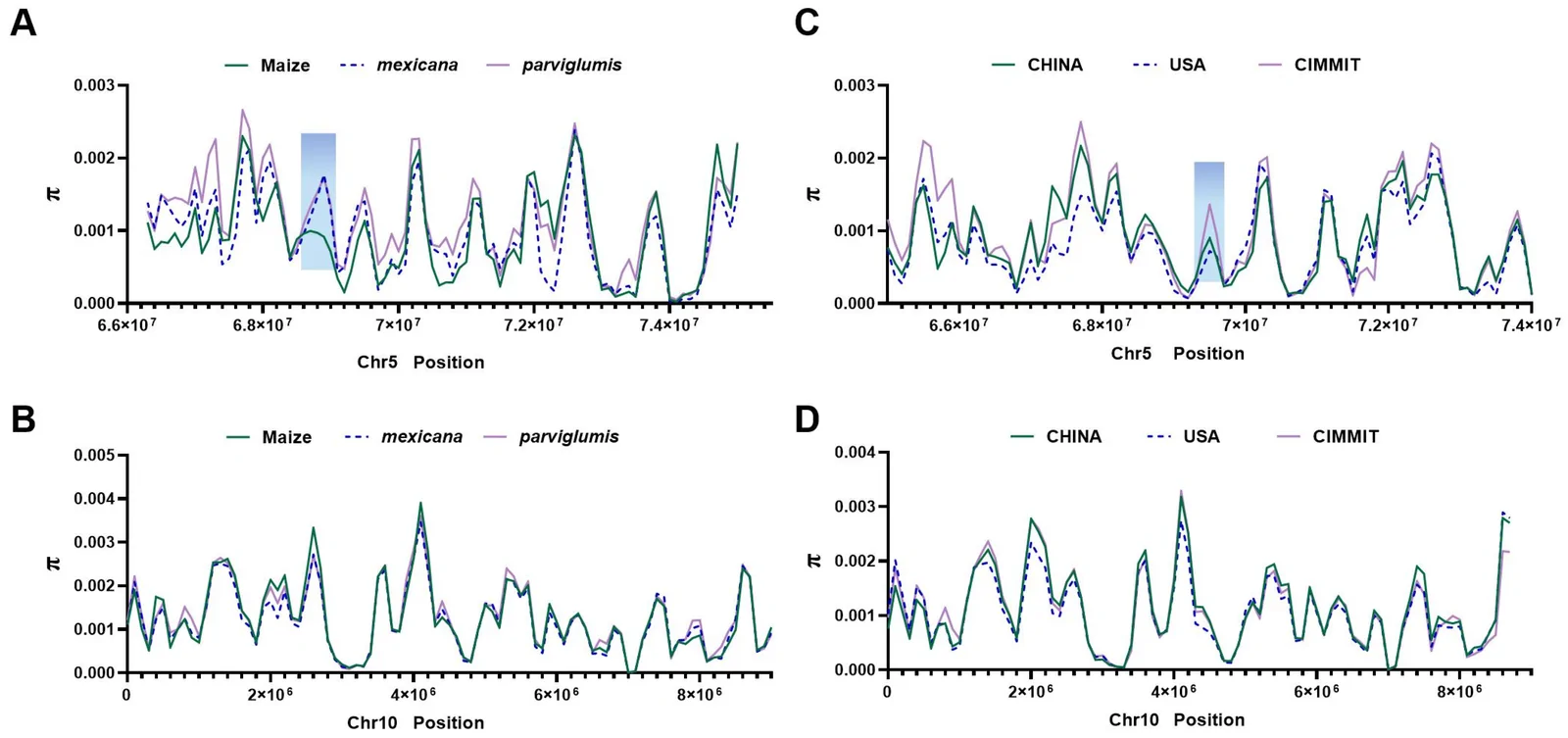
Selective sweep analysis of *Xt9* and *Zm00001d023417* across domestication and regional breeding. (**A**,**B**) Genetic differentiation between modern maize and two teosinte subspecies (*Zea mays* subsp. *parviglumis* and *Zea mays* subsp. *mexicana*) for *Xt9* (**A**) and *Zm00001d023417* (**B**). (**C**,**D**) Population differentiation among China, the United States, and CIMMYT germplasm for *Xt9* (**C**) and *Zm00001d023417* (**D**).

Selective sweep analyses were performed to investigate the evolutionary histories of the two prioritized candidate genes across maize domestication, breeding, and ecological adaptation (Figure 6). *Xt9* exhibited evidence of selection during multiple stages of maize improvement. During domestication, significant genetic differentiation was observed between modern maize and both *Zea mays* subsp. *parviglumis* and *Z. mays* subsp. *mexicana* (Figure 6A). Regional breeding analyses further identified significant differentiation between the U.S. breeding population and both the Chinese and CIMMYT populations, whereas no significant differentiation was detected between the Chinese and CIMMYT groups (Figure 6C). In contrast, no significant evidence of selection was detected for *Zm00001d023417*, the candidate xyloglucan xylosyltransferase, during either domestication or regional breeding, as no significant genetic differentiation was observed among the populations examined (Figure 6B,D).

**Figure 7:**
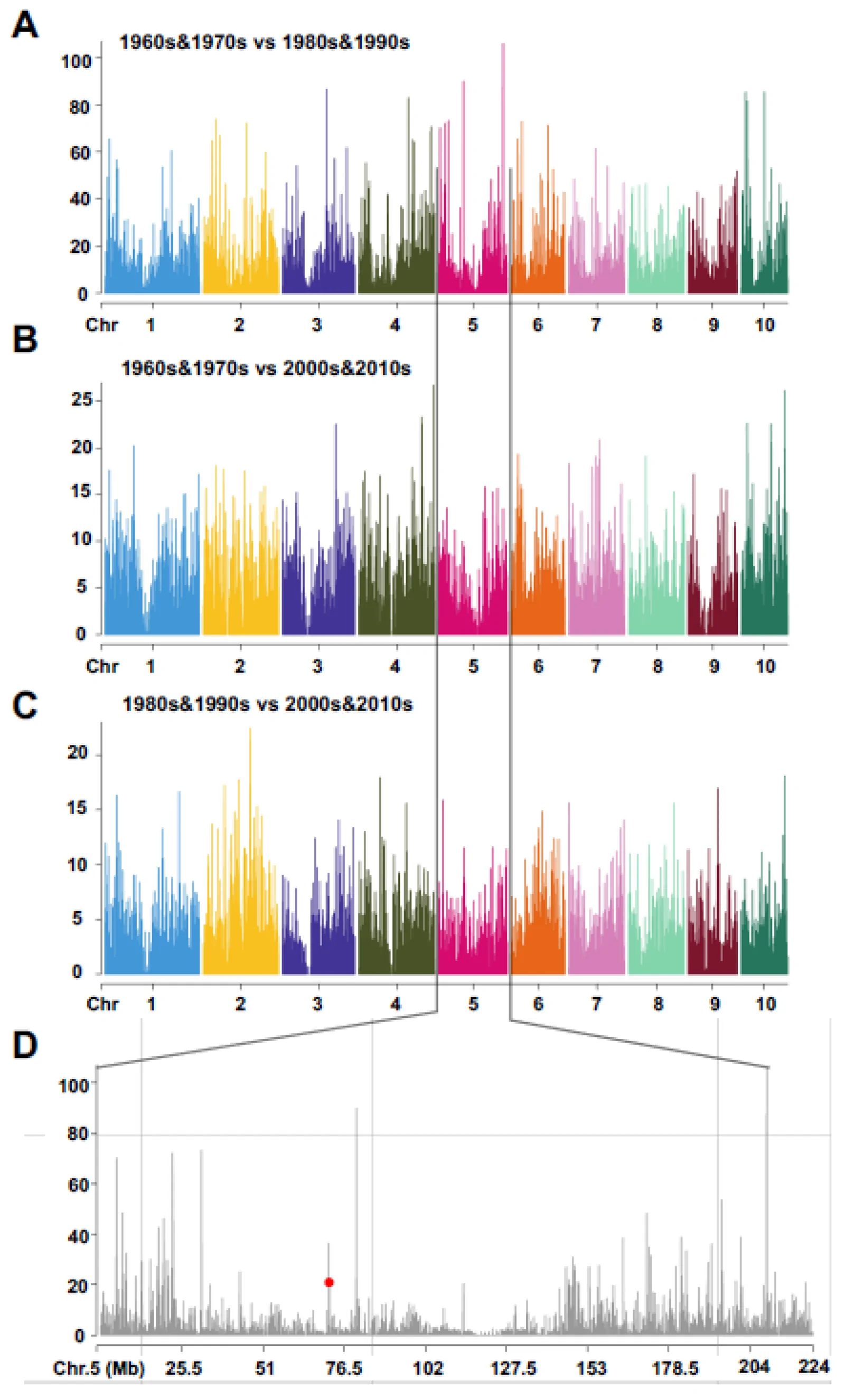
Temporal and ecological selective sweep analysis of *Xt9*. (**A**–**C**) Temporal selective sweep analysis across decadal breeding periods: 1960s–1970s vs. 1980s–1990s (**A**), 1960s–1970s vs. 2000s–2010s (**B**), and 1980s–1990s vs. 2000s–2010s (**C**). (**D**) Ecological ecotype analysis between temperate and tropical/subtropical populations based on XPCLR.

To investigate whether *Xt9* has been subject to artificial selection during modern breeding, we performed selective sweep analyses across three decadal subpopulations (1960s–1970s, 1980s–1990s, and 2000s–2010s) from the association panel. A significant selection signal at *Xt9* was detected between the 1960s–1970s and 1980s–1990s groups, whereas no significant differentiation was observed in comparisons involving the 2000s–2010s group (Figure 7A–C), suggesting that selective pressure on this locus may have diminished during more recent breeding periods. Comparisons between temperate and tropical/subtropical germplasm also revealed significant differentiation at *Xt9* (Figure 7D), consistent with selection during adaptation to temperate environments. Collectively, these results suggest that *Xt9* may have been subject to selection during maize domestication, modern breeding, and ecological adaptation. In contrast, *Zm00001d023417* showed no comparable evidence of selection despite its significant association with cell wall composition.

## 3. Discussion

The present study builds upon our previous GWAS of maize stalk cell wall composition by reanalyzing the same association panel [16] using a substantially denser marker dataset and a more comprehensive analytical framework. Increasing marker density from approximately 0.56 million to 10.77 million SNPs, together with the integration of six complementary GWAS models, greatly expanded the number of detectable loci and candidate genes. The present study adds to a growing body of work demonstrating that existing phenotypic datasets can continue to yield new biological insights when reanalyzed using improved genomic resources. Similar studies in soybean have shown that increasing marker density through genotype imputation or whole-genome resequencing enables the discovery of novel loci that were not detected in earlier GWAS while improving candidate gene resolution [27,28,29,30,31]. Furthermore, the addition of haplotype and selective sweep analyses provided multiple independent lines of evidence for candidate gene prioritization that were not included in the original study. These results illustrate how advances in genomic resources and analytical approaches can substantially increase the biological insight obtained from existing phenotypic datasets.

A major finding of this reanalysis was that the derived trait LIG/(CEL + HC) yielded substantially more significant associations than the three primary cell wall components. Cell wall formation is a highly coordinated biological process involving the synthesis, transport, and assembly of lignin and structural polysaccharides. Rather than acting independently, cellulose, hemicellulose, and lignin form an integrated network that collectively determines cell wall architecture, mechanical strength, and degradability [26,32]. Consequently, traits representing the relative balance among these components may better capture the underlying biological processes than measurements of individual polymers alone. Similar observations have been reported in forage quality studies, where the relative proportions of lignin and structural carbohydrates explain variation in digestibility more effectively than absolute component contents [33]. Together, these findings suggest that derived compositional indices may provide useful metrics for identifying loci underlying complex cell wall traits.

The integration of multiple complementary analytical approaches substantially strengthened candidate gene prioritization beyond that achievable through GWAS alone. Although the six GWAS models differed in the number of significant associations detected, several loci were consistently identified across multiple methods, increasing confidence that these represent robust genetic associations rather than model-specific artifacts. Functional enrichment analyses further demonstrated that many candidate genes participate in transferase activity, carbohydrate metabolism, and other biological processes associated with secondary cell wall formation. By combining cross-model support with functional annotation and haplotype analysis, the present study prioritized a small number of high-confidence candidate genes from hundreds of initial associations. This integrated strategy illustrates the value of combining complementary genomic and evolutionary analyses to improve candidate gene discovery for complex quantitative traits.

Among the prioritized candidates, *Zm00001d002476*, encoding a MYB58 transcription factor, represents a biologically compelling candidate because its Arabidopsis ortholog functions as a master regulator of lignin biosynthesis through activation of genes within the phenylpropanoid pathway [34]. Our findings both corroborate and extend previously reported QTL for maize cell wall composition. For instance, the genomic interval harboring *Xt9* on chromosome 4 overlaps with a major QTL for stalk lignin content and rind penetrometer resistance identified in multiple independent bi-parental populations [35,36]. Although haplotype analysis did not reveal significant phenotypic differences among allelic classes in the present association panel, this result does not necessarily preclude an important biological role. Genome-wide association studies identify genomic regions associated with phenotypic variation rather than causal polymorphisms, and functional variation at this locus may not be fully captured by the SNPs examined in the present study. In contrast, *Xt9*, encoding a glycosyltransferase, and *Zm00001d023417*, encoding a probable xyloglucan 6-xylosyltransferase, exhibited both biologically relevant functional annotations and significant haplotype effects. Glycosyltransferases are essential for the biosynthesis and modification of structural polysaccharides, whereas xyloglucan xylosyltransferases contribute to hemicellulose assembly and cell wall organization. Together, these findings suggest that natural variation affecting polysaccharide biosynthesis and remodeling contributes substantially to genetic variation in maize stalk cell wall composition.

Selective sweep analyses provided an additional layer of evidence for candidate gene prioritization by distinguishing loci that have experienced historical selection from those that have not. Among the prioritized genes, *Xt9* exhibited evidence of repeated selection during maize domestication, regional breeding, and ecological adaptation, suggesting that this locus has been repeatedly targeted during maize improvement. Given the importance of cell wall composition in determining stalk strength, biomass production, and adaptation to diverse environments, these signatures are consistent with historical selection acting on agronomically important traits influenced by cell wall architecture. In contrast, *Zm00001d023417* exhibited significant phenotypic associations but little evidence of historical selection. This contrast raises the possibility that favorable alleles at this locus remain underutilized in modern breeding populations and may therefore represent an additional source of genetic variation for future crop improvement. More broadly, these findings demonstrate how evolutionary analyses can complement association mapping by distinguishing candidate genes with contrasting breeding histories despite similar phenotypic effects.

Several limitations of this study should be acknowledged. First, although the expanded marker density substantially increased the number of statistically significant SNP–trait associations, this increase primarily reflects denser coverage of existing linkage disequilibrium (LD) blocks rather than improved mapping resolution per se. In regions of extensive LD, multiple correlated SNPs may tag the same underlying quantitative trait locus without enabling finer localization of the causal variant(s). Second, all associations reported here were identified within a single association panel and have not been independently validated in additional populations or through functional assays. Therefore, the candidate genes prioritized in this study should be regarded as statistically supported hypotheses requiring further experimental validation rather than as confirmed genetic determinants. Third, although the derived trait LIG/(CEL + HC) yielded the largest number of associations, whether this pattern reflects a genuine biological signal or the statistical properties of ratio traits remains to be determined and should therefore be interpreted with caution.

In summary, reanalysis of a well-characterized maize association panel using substantially improved genomic resources and an integrated analytical framework greatly expanded the resolution of association mapping and strengthened candidate gene prioritization. By combining ultra-high-density genotyping, complementary GWAS models, functional enrichment, haplotype analyses, and selective sweep analyses, this study identified candidate genes supported by multiple independent lines of evidence, with *Xt9* emerging as the strongest candidate through both functional and evolutionary analyses. Beyond advancing our understanding of the genetic architecture underlying maize stalk cell wall composition, this work illustrates the continued value of revisiting existing phenotypic datasets as genomic resources and analytical methods continue to improve.

## 4. Materials and Methods

### 4.1. Plant Materials and Phenotyping

The association mapping panel consisted of 341 diverse maize inbred lines, representing tropical/subtropical, stiff stalk (SS), non-stiff stalk (NSS), and mixed subpopulations. The germplasm originated from the International Maize and Wheat Improvement Center (CIMMYT), China, and the United States, with the CIMMYT lines consisting predominantly of tropical/subtropical germplasm [37]. Field trials were conducted across seven environments: Hainan and Yunnan in 2010; Hainan, Henan, and Yunnan in 2011; and Hainan and Yunnan in 2012. A randomized complete block design was used at each location without replication. Each line was planted in a single row (2.5 m in length) with 11 plants per row at a density of 60,000 plants/ha, with adjacent rows spaced 0.67 m apart. Stalk tissue samples were collected at maturity, and lignin (LIG), cellulose (CEL), and hemicellulose (HC) contents were determined using the Van Soest detergent fiber analysis method, as detailed in the original study [16]. In addition to these three primary traits, two derived compositional indices, cellulose-to-hemicellulose ratio (CEL/HC) and lignin-to-structural carbohydrate ratio [LIG/(CEL + HC)], were calculated from the published phenotypic data (Table S1). Mean phenotypic values across the seven environments were used for all subsequent analyses.

### 4.2. Genotype Data and Quality Control

The 341 inbred lines were previously genotyped using a high-density marker set comprising 10.77 million (10.77 M) high-quality single nucleotide polymorphisms (SNPs) with a minor allele frequency (MAF) threshold of 0.05, aligned to the B73 reference genome (RefGen_v4) [38,39]. Markers with a missing rate exceeding 10% or heterozygosity rate above 20% were excluded. SNPs were annotated based on their physical positions relative to gene models in the B73 RefGen_v4 annotation.

### 4.3. Genome-Wide Association Study (GWAS)

GWAS was performed using GAPIT v3 with six complementary models (GLM, MLM, MLMM, SUPER, FarmCPU, and BLINK). The first three principal components were included as fixed covariates to control for population structure, and the VanRaden kinship matrix was used where required by each model. For each model, a genome-wide significance threshold was determined by Bonferroni correction (α = 0.05/number of independent tests). Given the high marker density and widespread linkage disequilibrium (LD) across the maize genome, the effective number of independent tests was estimated using the simpleM method to eliminate the influence of LD between adjacent SNPs. A unified genome-wide significant threshold of 2.04 × 10^−6^ was adopted in the present study [40].

### 4.4. GO and KEGG Analysis

Candidate genes were converted to the B73 RefGen_v4 identifiers using MaizeGDB (https://maizegdb.org/; accessed on 5 July 2026). GO and KEGG enrichment analyses were performed using OmicShare Tools (https://www.omicshare.com/tools; accessed on 5 July 2026).

### 4.5. Candidate Gene Identification and Annotation

The genome-wide average LD decay distance was estimated by calculating pairwise squared correlation coefficients (R^2^) between SNPs across all chromosomes and fitting a locally weighted polynomial regression (LOESS) curve, with the critical r^2^ threshold set at 0.2. Based on the estimated LD decay distance, candidate regions were defined as genomic intervals extending 50 kb upstream and 50 kb downstream of each lead significant SNP (100 kb total). Genes located within these candidate regions were extracted from the B73 RefGen_v4 gene annotation (Zea mays AGPv4, Ensembl Plants release 49). Functional annotation of candidate genes was performed using InterProScan and BLASTP searches against the NCBI non-redundant (nr) protein database and the UniProtKB/Swiss-Prot database.

### 4.6. Haplotype and Linkage Disequilibrium Analysis

For high-confidence candidate genes, local LD patterns and haplotype block structures were characterized using LDBlockShow (https://github.com/BGI-shenzhen/LDBlockShow; accessed on 5 July 2026) [41]. Haplotype blocks were defined using the confidence interval method with default parameters. Only significant SNPs within the candidate gene region were used for haplotype construction. The phenotypic effects of different haplotypes on the five cell wall-related traits were evaluated using ANOVA followed by pairwise comparisons with two-sided Student’s *t*-tests. Haplotype–phenotype associations were visualized using boxplots generated with the R package ggplot2 and Origin 2024.

### 4.7. Selective Sweep and Population Differentiation Analysis

To assess whether candidate loci had been subject to selection during maize domestication and improvement, we performed population differentiation analysis using publicly available genotype data from diverse germplasm resources. The fixation index (FST) was calculated to quantify genetic differentiation between subgroups defined by geographic origin (Chinese, United States, and Mexican inbred lines) and by ecological adaptation (tropical/subtropical versus temperate germplasm) as described previously [42]. FST values were computed in sliding windows of 25 kb with a step size of 20 kb across candidate QTL intervals. Genomic regions with FST values exceeding the top 5% empirical distribution were considered to be under selection, and candidate genes within these regions were flagged as potential targets of artificial or natural selection during maize evolution and breeding. XP-CLR analysis was performed in R using genome-wide SNP data to identify genomic regions potentially subjected to selection. Pairwise comparisons were conducted between temperate and tropical/subtropical germplasm, and regions showing elevated XP-CLR scores were considered candidate selective-sweep regions.

### 4.8. Statistical Analyses and Software

All GWAS analyses were conducted using GAPIT v3 implemented in R (version 4.2.0). Phenotypic data processing, heritability estimation, and descriptive statistics were performed using the R packages lme4 (version 1.1-35.5), rrBLUP (version 4.6.3), and psych (version 2.4.12). Haplotype and population genetic analyses (LD decay, FST) were carried out using PopLDdecay, VCFtools (version 0.1.16), and custom R and Python scripts. Visualization was performed using ggplot2 (version 3.5.1), CMplot (version 4.5.1), Origin 2024, and Circos (version 0.69-9).

## Data Availability

Data are contained within the article or Supplementary Material.

## Supplementary Materials

The following supporting information can be downloaded at: https://www.mdpi.com/article/10.3390/plants15172696/s1, Supplementary Figure S1: Quantile-quantile (Q-Q) plots for all model–trait combinations. (A–F) Q-Q plots for BLINK, FarmCPU, GLM, MLMM, SUPER, and MLM models, respectively, demonstrating adequate control of genomic inflation with no spurious associations introduced by population structure or kinship; Supplementary Figure S2: Manhattan plots of genome-wide association signals for cell wall components. (A–F) Manhattan plots for BLINK, FarmCPU, GLM, MLMM, SUPER, and MLM models, respectively, showing genome-wide significant associations for lignin, cellulose, hemicellulose, CEL/HEM ratio, and LIG/(CEL + HC) ratio across seven environments. The dashed horizontal line indicates the genome-wide significance threshold; Table S1:The phenotypic data of 341 maize inbred lines used in this study; Table S2: Descriptive statistics of stalk cell wall component traits evaluated in the maize association mapping population; Table S3: Significant loci identified by six GWAS models; Table S4: Co-localization of candidate loci between the previous (0.56 SNPs) and current (10.77M SNPs) GWAS for maize stalk cell wall composition; Table S5: Top 30 enriched Gene Ontology terms among genes associated with significant GWAS SNPs; Table S6: Top 30 enriched KEGG Pathway among genes associated with significant GWAS SNPs; Table S7: Haplotype information of Zm00001d014965; Table S8: Haplotype information of Zm00001d023417.
