# Supplementary material for: High-Density Genome-Wide Association Mapping Identifies Candidate Loci Associated with Maize Stalk Cell Wall Composition
Source: Plants (Basel). 2026 Sep 2;15(17):2696. doi: 10.3390/plants15172696 (PMC13568257; doi:10.3390/plants15172696)
Supplement: Supplementary file 1 [file plants-15-02696-s001.zip › Supplementary Figures.pdf]

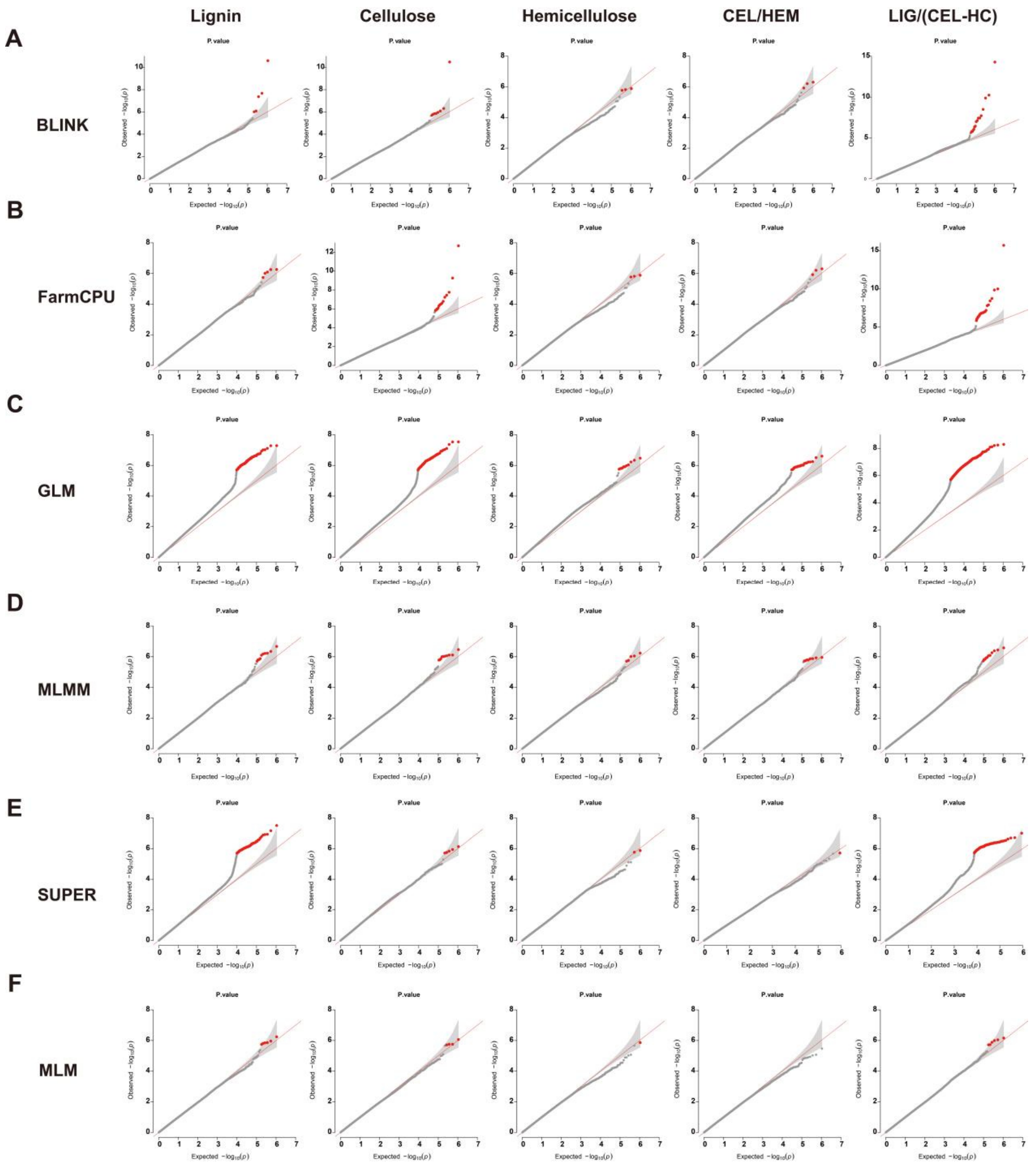

Supplementary Figure S1. Quantile-quantile (Q-Q) plots for all model–trait combinations. (A–F) Q-Q plots for BLINK, FarmCPU, GLM, MLMM, SUPER, and MLM models, respectively, demonstrating adequate control of genomic inflation with no spurious associations introduced by population structure or kinship.

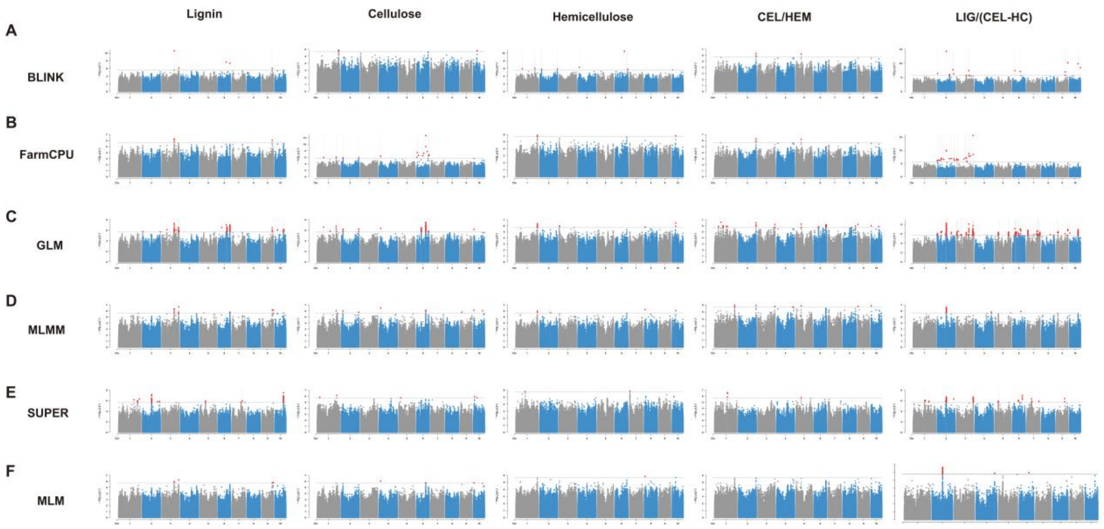

Supplementary Figure S2. Manhattan plots of genome-wide association signals for cell wall components. (A–F) Manhattan plots for BLINK, FarmCPU, GLM, MLMM, SUPER, and MLM models, respectively, showing genome-wide significant associations for lignin, cellulose, hemicellulose, CEL/HEM ratio, and LIG/(CEL+HC) ratio across seven environments. The dashed horizontal line indicates the genome-wide significance threshold.
